# Exploring Information Available to and Used by Physicians on Antibiotic Use and Antibiotic Resistance in Jordan

**DOI:** 10.3390/antibiotics10080963

**Published:** 2021-08-11

**Authors:** Reema A. Karasneh, Sayer I. Al-Azzam, Mera A. Ababneh, Iman A. Basheti, Ola Al-Azzeh, Sarah Al Sharie, Barbara R. Conway, Mamoon A. Aldeyab

**Affiliations:** 1 Department of Basic Medical Sciences, Faculty of Medicine, Yarmouk University, Irbid 21163, Jordan; reema.karasneh@yu.edu.jo; 2 Department of Clinical Pharmacy, Faculty of Pharmacy, Jordan University of Science and Technology, Irbid 22110, Jordan; salazzam@just.edu.jo (S.I.A.-A.); mababneh@just.edu.jo (M.A.A.); 3 Faculty of Pharmacy, Applied Sciences Private University, Amman 11931, Jordan; dr_iman@asu.edu.jo; 4 Department of Pharmacy Practice, College of Pharmacy, King Saud Bin Abdulaziz University for Health Sciences, Riyadh 11481, Saudi Arabia; azzeho@ksau-hs.edu.sa; 5 Faculty of Medicine, Yarmouk University, Irbid 21163, Jordan; sarahalsharie2000@gmail.com; 6 Department of Pharmacy, School of Applied Sciences, University of Huddersfield, Huddersfield HD1 3DH, UK; b.r.conway@hud.ac.uk; 7 Institute of Skin Integrity and Infection Prevention, University of Huddersfield, Huddersfield HD1 3DH, UK

**Keywords:** antibiotics use, antibiotics resistance, physicians, information, national action plans

## Abstract

Evidence based information sources for physicians are needed for informed antibiotic prescribing practices. The aim of this study was to explore physicians’ preferred sources of information and evaluate physicians’ awareness of available information and initiatives on prudent antibiotic prescribing in Jordan. A cross-sectional study was conducted utilizing an online questionnaire and included physicians (*n* = 409) from all sectors and specialties in Jordan. Published guidelines (31.8%), the workplace (25.7%), colleagues or peers (20.0%), group or conference training (18.3%), and the medical professional body (18.1%) were the main sources of information about avoiding unnecessary antibiotic prescribing, with the influence of these sources on changing prescribers’ views being 34.7%, 17.1%, 11%, 13.4%, and 7.6%, respectively. One-third of physicians (33.7%) reported no knowledge of any initiatives on antibiotic awareness and resistance. Regarding awareness of national action plans on antimicrobial resistance, 10.5%, 34%, and 55.5% of physicians were aware, unaware, and unsure of the presence of any national action plans, respectively. Physicians showed interest in receiving more information on resistance to antibiotics (58.9%), how to use antibiotics (42.2%), medical conditions for which antibiotics are used (41.3%), prescribing of antibiotics (35.2%), and links between the health of humans, animals, and the environment (19.8%). The findings can inform interventions needed to design effective antimicrobial stewardship, enabling physicians to prescribe antibiotics appropriately.

## 1. Introduction

The introduction of antibiotics has been considered a huge revolution in the medical field, helping to reduce mortality rates and countless complications of infectious diseases [1,2]. Despite the remarkable benefits of antibiotics, if taken inappropriately, they can lead to issues with resistance [3,4,5]. Therefore, regulation of antibiotic prescription and dispensation is required [6]. In Jordan, current regulations state that antimicrobials can only be dispensed by prescription, and only physicians and dentists have the authority to prescribe antibiotics [7]. However, this is not well controlled, and antibiotics can still be dispensed without a prescription as per patient request, thus worsening the problem of antimicrobial resistance [8,9]. Furthermore, this issue may be exacerbated by the misuse and overuse of antibiotics caused by inappropriate prescribing by physicians without adequate supportive clinical information [3,10,11,12,13].

Over time, physicians have been considered as the gatekeepers for pharmaceutical prescriptions, including antibiotics, and the place where patients lay their trust to receive the most optimal healthcare available [14]. The field of medical knowledge has been growing expeditiously over the years [15], and as this field has grown bigger, the number of sources supplying medical information for patients, healthcare givers, and physicians has increased [16]. As a result of the massive number and huge variation of available medical information sources, physicians differ in their preferred sources of information [17,18].

Doctors can receive information through a number of sources, such as published articles in medical journals, meetings, conferences, the internet and social media, and most importantly through clinical practice guidelines [19,20,21,22,23]. Various factors can affect the choice of physicians regarding used sources of information such as age, past training, and specialty [24] in addition to physicians’ familiarity and previous expertise with information systems and sources [22]. Moreover, convenience of access and the availability and credibility of data supplied by sources can play major roles in the choice of preferred information sources among doctors [19,25,26]. In order for a medical information source to be valid and reliable, it should reference previously published literature and be constantly updated with the newest medical guidelines, interventions, and achievements [27,28].

Even though there is a wide range of research about physicians’ sources of information, until now, the most used, reliable, and suitable sources of information for physicians in prescribing antibiotics have remained unclear [29]. The use of trustworthy information sources such as defined treatment guidelines is needed in order for physicians to prescribe suitable antibiotics, as these sources have a strong impact on clinical practices, leading to better understanding and more effective management of various medical encounters [30,31]. Prudent use of antibiotics can also be facilitated through information provided by international and national initiatives and action plans such as that undertaken by the World Health Organization (WHO) [32,33,34,35]. Furthermore, immediately available sources are now considered essential, particularly with the emergence of smartphones and social media [36,37,38]. Therefore, in this study we aimed to evaluate physicians’ preferred sources of information and physicians’ awareness of available information and initiatives on prudent antibiotic prescribing practices in Jordan.

## 2. Results

The study included 409 physicians; 74.3% were male, 76.5% were aged between 25 and 35, and 70.4% were within the first 5 years of practice (Table 1).

Half of included physicians were generalists practicing in hospitals. Physicians’ management of patients with infections was mainly based on clinical practice guidelines (66.0%), their previous clinical experience (46.9%), and continuing education training courses (39.6%) (Table 2).

More than half of participants (59.4%) reported that they had received information about avoiding unnecessary prescribing of antibiotics in the last 12 months, and that this information contributed to changing their views, as 97.1% of this subset of participants did change their practice (Table 3).

**Figure 1:**
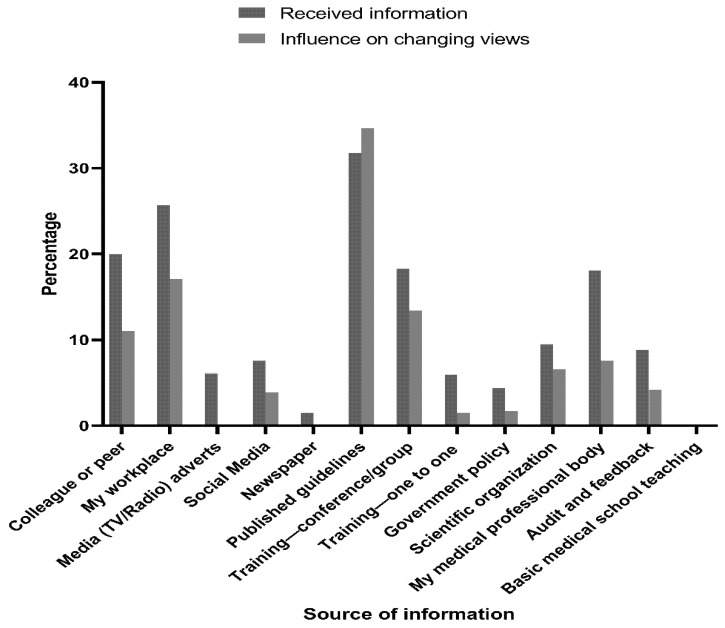
Sources of information about avoiding unnecessary prescribing antibiotics and their influence on changing physicians’ views (*n* = 409).

The main sources of information about avoiding unnecessary prescribing of antibiotics were published guidelines (31.8%) and the workplace (25.7%), which had the highest impacts on changing physician’s views (34.7% and 17.1%, respectively). Other sources included colleagues or peers (20.0%), group or conference training (18.3%), and the medical professional body (18.1%) (Figure 1). Interestingly, social media was found to have more influence on changing views (3.9%) and providing more information about avoiding unnecessary prescribing antibiotics (7.6%) than one-to-one training, basic medical school teaching, other media sources (e.g., TV, newspaper), or government policy (Figure 1). In fact, social media networks were found to be used for professional activities among 88.3% (*n* = 361) of physicians in this study, with Facebook being the most popular social media network (*n* = 280, 68.5%), followed by Instagram (*n* = 84, 20.5%), Google+ (*n* = 72, 17.6%), YouTube (*n* = 48, 11.7%), LinkedIn (*n* = 32, 7.8%), and Twitter (*n* = 31, 7.6%).

Regarding awareness of national action plans on antimicrobial resistance, 10.5% (*n* = 43), 34% (*n* = 139), and 55.5% (*n* = 227) of physicians were aware, unaware, and unsure of presence of any national action plans, respectively. Moreover, one-third of physicians in this study (33.7%) reported that they were not aware of any initiative focusing on antibiotic awareness and resistance. However, physicians claimed that they were aware of either conferences and events on tackling antibiotic resistance (27.6%), national or regional guidelines on management of infections (24.6%), or awareness raising by professional organizations (23.9%) (Table 4).

Physicians showed interest in receiving more information on several related topics, including resistance to antibiotics (58.9%, *n* = 241), how to use antibiotics (42.2%, *n* = 173), medical conditions for which antibiotics are used (41.3%, *n* = 169), prescription of antibiotics (35.2%, *n* = 144), and links between the health of humans, animals, and the environment (19.8%, *n* = 81).

## 3. Discussion

In this study, we investigated information available to and used by physicians in relation to antibiotic use and decision making along with the influence of this information on antibiotic prescribing views and behavior. The national action plan, for instance, promotes investments in AMR activities through providing evidence based antibiotic prescribing guidelines to all sectors and developing guidance for information materials [39]. Therefore, in this study we assessed the use of recent guidelines, continuing education of practitioners, and awareness regarding national efforts to counteract the emerging antibiotic resistance issue.

While more than half of the physicians surveyed indicated that they had been educated about prudent antibiotic use during the last year. A study conducted by Deuster and his colleagues indicated that implementation of guidelines and educating practitioners resulted in a significant increase in proper antibiotics use [31]. The majority of our physicians admitted that the information provided to them guided their prescribing practices, which is also similar to the results of another study that was conducted in Ireland with a positive effect toward managing antimicrobial resistance [40,41].

Studies have shown that there are multiple factors that may contribute to antibiotic prescribing behavior among practitioners, including physicians’ attitude and knowledge, the patient–physician relationship, physicians’ experience, and other psychological factors [42,43,44,45]. While studying the factors that affect the prescribers’ intentions toward prescribing antibiotics, physicians’ specialty was among the most important contributors. The least intention to prescribe antibiotics was observed among pediatricians; however, more antibiotic prescriptions were provided to patients by family physicians and emergency physicians [46,47,48]. This may be attributed to differences in the setting of patient evaluation and compliance to patient follow-up [47].

Our results showed that the practitioners relied mostly on published guidelines for antibiotic prescribing, which is among the most important strategies to enhance prudent antibiotic prescribing behavior [49,50,51]. Generally, the implementation of guidelines and antimicrobial stewardship initiatives to direct prudent antibiotic prescribing are key elements in enhancing the appropriate use of antibiotics [41,44,49,52,53,54].

The practitioners showed a high interest in social media to receive updated information. This finding was similar to results of a systematic review that investigated a large variety of studies to conclude that 79.8% of the studies supported the educational contribution of social media networks even in healthcare domains [55]. Although all ages are increasingly using social media, differences between residents and physicians have been observed [56,57]. Similar to results of our study, where our practitioners showed the highest interest in Facebook, Pollock and colleagues’ study found that Facebook is the most used networking site when it comes to getting information [55]. Although those social and professional networks readily accessible, there are associated risks, which may include unreviewed content, misleading information, and unauthorized medical advice [58,59]. The role of antimicrobial stewardship in managing prudent antimicrobial use worldwide is well known, but surprisingly, more than 55% of practitioners were not aware of any local plans focused on decreasing antibiotic resistance and toward advocating more rational antibiotic use [54,60,61]. In contrast to these findings, another study found that pharmacists in Jordan had a high level of awareness about antibiotic resistance and the role antimicrobial stewardship [62]. Contrary to the view that initiatives around antibiotic stewardship are of minimal benefit in minimizing unnecessary antibiotic use, the positive effects of antibiotic stewardship on reducing antibiotic use have been reported in Jordan [63]. Therefore, it has been recommended to tailor efforts toward the implementation of antibiotic stewardship broadly in Jordan in order to enhance antibiotic utilization. This was also supported in several studies conducted in the Middle East that suggested more effective awareness strategies [63,64].

The study had the strength of using a robustly validated questionnaire; however, selection bias may have been introduced, as responses were obtained using an online survey through social media. Despite this, selection bias is less likely to have impacted our results, particularly with the observed increase in the use of smartphones among physicians [65,66]. Young age may also have impacted our results; however, it has been evidenced that the use of smartphones and social media are prevalent across all ages and professions [57,67,68].

In conclusion, the findings of this study can inform a more effective role of antimicrobial stewardship in Jordan with tailoring of efforts to increase awareness regarding antimicrobial stewardship efforts, roles, and initiatives. Encouraging physicians to refer to local guidelines and bacterial resistance status periodically to get updated about the status in Jordan is required. Moreover, utilization of social media for health educational purposes should be promoted to reach more healthcare providers; however, this may require the published content to be reviewed by a professional body.

## 4. Materials and Methods

An online survey was utilized to conduct a cross-sectional study targeting physicians from all sectors in Jordan. Data was collected using a web-based survey software (Google Forms) by sending a link to physicians by direct institutional emails and/or social media through various Facebook pages for Jordanian physicians, personal messages, and postings in Jordanian physician-focused online forums. The link included a brief description of the study and its aims and objectives in addition to an invitation to participate. Survey questions appeared only after obtaining the physician’s consent to participate by choosing the “accept” option. The two options “required” and “limit to one response” were applied to survey questions for validation. Participants were informed that their participation was voluntary and that their responses would be anonymized and treated as confidential.

A 10 min online questionnaire was adapted from the questionnaire developed and tested by the European Center for Disease Prevention and Control (ECDC) [69]. (Questionnaire S1—Supplementary Material). The questionnaire was written in two languages: English, as it is the medium for physicians’ practice in Jordan, and Arabic, as it is the first language in Jordan. The translation was validated following a forward–backward procedure, and participants were able to choose their preferred language for participation. The final version of the questionnaire was further tested for content and face validity by experts in the field who provided their constructive suggestions and feedback, particularly on the Arabic section of the questionnaire. Pilot testing was also conducted, and these data were not included in the final sample.

The survey consisted of 16 questions. The first section included 6 questions about sociodemographic variables including age, gender, role, years of expertise, and practice place and setting. The second section included 2 questions on used social media and used resources for professional activities and the management of infections; the option to choose all that apply was available for these 2 questions. The third section included 5 questions about whether physicians had received information on avoiding unnecessary prescribing of antibiotics and if this information contributed to changing their views or practice. Physicians were also asked about sources of information about avoiding unnecessary prescribing of antibiotics and the influence of these sources on changing physicians’ views. Awareness of any national action plans and initiatives on antimicrobial resistance were assessed in the last section; selecting more than one option was possible for respondents for initiatives awareness question. In addition, physicians were asked about topics related to antibiotics use that they would like to receive more information on.

The sample size was determined using the Raosoft sample size calculator based on a margin of error of 5%, confidence level of 95%, population size of 28,000 (registered physicians in Jordan), and a response distribution of 50%. The calculated sample size was 379. Figures were produced using GraphPad Prism. Collected data was analyzed using the IBM SPSS (Statistical Package for the Social Sciences) version 24.0 software. Results were displayed using frequencies and percentages.

## Figures and Tables

**Table 1:** Sociodemographic characteristics (*n* = 409).

Variable	Category	Frequency	Percentage %
Gender	Female	105	25.7
	Male	304	74.3
Age (years)	25–35	313	76.5
	36–55	60	14.7
	≥56	36	8.8
Role of specialist	Specialist	182	44.5
	Generalist	200	50.4
	Academia/Research	21	5.1
Years of practice (*n*)	0–5	288	70.4
	6–15	59	14.4
	≥16	62	15.2
Place of practice	Public Clinic	45	11.0
	University/Research institute	22	5.1
	Hospital	239	58.4
	Private Clinic	103	25.2
Governorate (region)	North	192	46.9
	Middle	191	46.7
	South	26	6.4

**Table 2:** Resources used for management of patients with infections.

In the Management of Infections, Which of These Do You Use Regularly?	Frequency	Percentage %
Clinical practice guidelines	270	66.0
Documentation from the pharmaceutical industry	46	11.2
Medical representatives from industry	29	7.1
Previous clinical experience	192	46.9
Continuing education training courses	162	39.6
Infection specialists	82	20.0
Scientific journals	45	11.0
Professional resources/publications	67	16.4
Social media	33	8.1
None of the above	7	1.7

**Table 3:** Number of respondents (%) who received information on avoiding unnecessary prescribing of antibiotics and, of those, the number (%) reporting that the information contributed to changing their views or practice.

Questions	Yes *n* (%)	No *n* (%)	Unsure *n* (%)
In the last 12 months, do you remember receiving any information about avoiding unnecessary prescribing of antibiotics? (*n* = 409)	243 (59.4)	149 (36.4)	17 (4.2)
Did the information contribute to changing your views about avoiding unnecessary prescribing of antibiotics? (*n* = 243)	243 (100.0)	0 (0.0)	0 (0.0)
On the basis of the information you received, have you changed your practice on prescribing antibiotics? (*n* = 243)	236 (97.1)	2 (0.8)	5 (2.1)

**Table 4:** Awareness of initiatives on antibiotic awareness and resistance.

Items	Frequency	Percentage (%)
I am not aware of any initiatives	138	33.7
TV or radio advertising for the public	78	19.1
World Antibiotic Awareness Week	42	10.3
Conferences/events focused on tackling antibiotic resistance	113	27.6
National or regional posters or leaflets on antibiotic awareness	72	17.6
Awareness raising by professional organizations	98	23.9
Toolkits and resources for healthcare workers	80	19.6
National or regional guidelines on management of infections	101	24.6
Newspaper (national) articles on antibiotic resistance	38	9.3

## Data Availability

Data available on reasonable request and in line with permission approval processes from the Jordan University of Science and Technology.

## Supplementary Materials

The following are available online at https://www.mdpi.com/article/10.3390/antibiotics10080963/s1, Questionnaire S1. Survey of physicians’ sources of information about antibiotic use and antibiotic resistance.
