# Supplementary material for: Exploring Information Available to and Used by Physicians on Antibiotic Use and Antibiotic Resistance in Jordan
Source: Antibiotics (Basel). 2021 Aug 11;10(8):963. doi: 10.3390/antibiotics10080963 (PMC8389019; doi:10.3390/antibiotics10080963)
Supplement: Supplementary file 1 [file antibiotics-10-00963-s001.zip › antibiotics-1318901-supplementary.pdf]

Survey of physicians' sources of information about antibiotic use and antibiotic resistance

Dear Respondent,
You are invited to complete the following survey of physicians about their knowledge and attitudes about antibiotic use and antibiotic resistance.
Researchers from UK, Jordan University of Science and Technology, and Yarmouk University are seeking responses from physicians. We would really value you completing the survey that will take 5 to 10 minutes to complete. It includes predominantly multiple-choice questions.
Please feel free to cascade the link of the survey widely to colleagues.

In which language would you prefer to complete this survey? بأيّ لغة تفضل اتمام الاستبيان ؟
English
Arabic

- **Demographic Section**

1. Please specify in which governorate you practice
Amman
Irbid
Salt
Zarqa
Madaba
Jerash
Ajloun
Mafraq
Karak
Tafilah
Ma'an
Aqaba

2. What is your predominant role? (i.e.>50% of your time)?
Generalist
Specialist
Academia/ Research

3. Where do you predominantly practice? (i.e. >50% of your time):
Hospital (any hospital type)
University (as an Academic) or research institute

Public clinic
Private clinic

4. How many years have you been practicing in your current profession?
0-2 years
3-5 years
6-10 years
11-15 years
16-20 years
21-25 years
>25 years

5. What is your age?
24-35 years
36-45 years
46-55 years
56-65 years
>66 years

6. What gender do you identify with?
Male
Female

➢ Used resources for professional activities Section

7. Which of the following social media networks do you mainly use for professional activities? (Choose all that apply)
Twitter
Facebook
LinkedIn
Google+
YouTube
Instagram
I do not use social media
Others_____________________________________

8. In the management of infections, which of these do you use regularly? (Choose all that apply)
Clinical practice guidelines
Documentation from the pharmaceutical industry
Medical representatives from industry
Previous clinical experience
Continuing education training courses
Infection specialists
Scientific journals

Professional resources/publications
Social media
None of the above
I do not know
Others: ____

➢ Sources of information about avoiding unnecessary prescribing of antibiotics Section

9. In the last 12 months, do you remember receiving any information about avoiding unnecessary prescribing of antibiotics?
Yes
No
Unsure

10. How did you first get this information about avoiding unnecessary prescribing of antibiotics? (Select all that apply).
Colleague or peer
My workplace
Media (TV/Radio) adverts
Social Media
Newspaper
Published guidelines
Training - conference/group
Training - one to one
Government policy
Scientific organization
My medical professional body
Audit and feedback
Others: ____

11. Did the information contribute to changing your views about avoiding unnecessary prescribing of antibiotics?
Yes
No
Unsure

12. Which source(s) of information has had the most influence on changing your views? Select no more than 2.
Colleague or peer
My workplace
Media (TV/radio) adverts
Social media
Newspaper

Published guidelines
Training - conference/group
Training - one to one
Government policy
Scientific organization
My medical professional body
Audit and feedback
Others: ____

13. **On the basis of the information you received, have you changed your practice on prescribing of antibiotics?**
Yes
No
Unsure

➢ Awareness of initiatives and national action plans on antimicrobial resistance Section

14. **What initiatives are you aware of which focus on antibiotic awareness and resistance? Select all that apply**
TV or Radio advertising for the public
Toolkits and resources for healthcare workers
National or regional guidelines on management of infections
Awareness raising from professional organizations
Conference/Events focused on tackling antibiotic resistance
National or regional posters or leaflets on antibiotic awareness
Newspaper (national) articles on antibiotic resistance
World Antibiotic Awareness Week
I am not aware of any initiatives

15. **Does your country have a national action plan on antimicrobial resistance?**
Yes
No
Unsure

16. **On which topics would you like to receive more information? (Choose all that apply)**
Resistance to antibiotics
How to use antibiotics
Medical conditions for which antibiotics are used
Prescription of antibiotics
Links between the health of humans, animals and the environment
None
Others: ____________________________
